# The Pore-Forming α-Toxin from *Clostridium septicum* Activates the MAPK Pathway in a Ras-c-Raf-Dependent and Independent Manner

**DOI:** 10.3390/toxins7020516

**Published:** 2015-02-10

**Authors:** Anjana Chakravorty, Milena M. Awad, Jackie K. Cheung, Thomas J. Hiscox, Dena Lyras, Julian I. Rood

**Affiliations:** Department of Microbiology, Monash University, Clayton, VIC 3800, Australia; E-Mails: achak27@gmail.com (A.C.); milena.awad@monash.edu (M.M.A.); jackie.k.cheung@monash.edu (J.K.C.); thomas.hiscox@monash.edu (T.J.H.); dena.lyras@monash.edu (D.L.)

**Keywords:** *Clostridium septicum*, mitogen-activated protein kinases, α-toxin, pathogenesis, pore-forming toxins, gas gangrene

## Abstract

*Clostridium septicum* is the causative agent of atraumatic gas gangrene, with α-toxin, an extracellular pore-forming toxin, essential for disease. How *C. septicum* modulates the host’s innate immune response is poorly defined, although α-toxin-intoxicated muscle cells undergo cellular oncosis, characterised by mitochondrial dysfunction and release of reactive oxygen species. Nonetheless, the signalling events that occur prior to the initiation of oncosis are poorly characterised. Our aims were to characterise the ability of α-toxin to activate the host mitogen activated protein kinase (MAPK) signalling pathway both *in vitro* and *in vivo*. Treatment of Vero cells with purified α-toxin activated the extracellular-signal-regulated *kinase* (ERK), c-Jun *N*-terminal *kinase* (JNK) and p38 arms of the MAPK pathway and stimulated the release of TNF-α in a dose-dependent manner. Studies using inhibitors of all three MAPK components suggested that activation of ERK occurred in a Ras-c-Raf dependent manner, whereas activation of JNK and p38 occurred by a Ras-independent mechanism. Toxin-mediated activation was dependent on efficient receptor binding and pore formation and on an influx of extracellular calcium ions. In the mouse myonecrosis model we showed that the MAPK pathway was activated in tissues of infected mice, implying that it has an important role in the disease process.

## 1. Introduction

*Clostridium septicum* is a Gram-positive, spore-forming anaerobic rod that is present in the environment and in the gastrointestinal tract of humans and animals [1,2]. *C. septicum* is the causative agent of both traumatic and atraumatic gas gangrene and disease is usually initiated when wounds become contaminated with either vegetative cells or spores. In atraumatic gas gangrene, infection occurs at distal sites or when there is a breach in the gastrointestinal barrier [3]. Infection primarily occurs in severely immunocompromised hosts; *C. septicum* is a major cause of infection in adults with severe hematological malignancies and colorectal cancer, as well as in children with severe neutropenia [4].

The major virulence factor produced by *C. septicum* is α-toxin, a β-barrel pore-forming cytolysin [5,6]. Mutagenesis studies have shown that α-toxin is the primary virulence factor in *C. septicum*-mediated disease since deletion of the α-toxin structural gene (*csa*) renders the resultant strain avirulent in a mouse myonecrosis model [7]. Although infection with *C. septicum* mimics some of the features seen in *Clostridium perfringens*-mediated gas gangrene, in particular, the absence of polymorphonuclear leukocytes (PMNs) within the site of infection [7], the mechanism by which this process occurs in *C. septicum* infections is different and is poorly understood.

α-toxin has structural similarity to aerolysin from *Aeromonas hydrophilia* [5]. It is initially secreted as inactive 46.5 kDa protoxin monomers that are capable of binding to glycosylphosphatidylinositol (GPI)-anchored proteins [8,9] in lipid rafts [10], via a tryptophan-rich motif located in the *C*-terminal region [11,12]. Since GPI receptors are expressed on the surface of many cells, α-toxin has broad host cell specificity, although the proteins attached to the GPI-anchors have been shown to provide some specificity [8,13]. After binding, the monomeric subunits are cleaved by host cell proteases such as furin, which facilitates oligomerisation as a result of increased access to sites involved in protein-protein interactions [5,6]. Following oligomerisation, α-toxin forms heptameric ion-permeable pores on the host cell surface [5,6]. These pores promote the unregulated entry of extracellular ions, including calcium, into intoxicated cells and may eventually lead to cell lysis.

We have shown that *C. septicum* α-toxin-mediated pore formation causes an influx of extracellular calcium into intoxicated C2C12 mouse myoblast cells and consequently triggers downstream signalling events. These events include activation of the calpain-cathepsin pathway, disruption of lysosomal and mitochondrial integrity, reactive-oxygen species (ROS) production and HMGB-1 nuclear translocation, all of which eventually conspire to induce cellular oncosis of the intoxicated cell [14]. Other studies have shown that recombinant α-toxin forms large diffusion pores in lipid bilayers, which in cellular systems leads to rapid potassium ion efflux, ATP depletion, necrosis and cell death [15]. Accordingly, it is likely that the mechanism by which α-toxin induces cell death is complex and most likely multifactorial. This complexity raises questions regarding the signalling events triggered within α-toxin-intoxicated cells and which lead to their entry into an oncotic pathway.

The mitogen activated protein kinase (MAPK) pathway is one of the major pathways activated by cells following infection and intoxication [16]. This pathway involves the activation of a series of signals that are initiated by cellular contact with numerous stimuli and involves a series of phosphorylation events mediated by specific kinases. A MAP-kinase-kinase-kinase (MAPKKK) phosphorylates a MAP-kinase-kinase (MAPKK), which then phosphorylates a MAP-kinase (MAPK), with MAPK activation requiring both tyrosine and threonine phosphorylation [16,17,18]. The MAPK pathway is composed of three main subsets of kinases: specifically, extracellular-signal-regulated kinase (ERK) 1/2 (p42/p44), c-Jun N-terminal kinase (JNK) 1/2 (SAPK) and p38. A stimulus may specifically activate one or all of these kinase subsets and activation of one pathway may also cause the activation or deactivation of the other pathways [16,17,18]. The end result of this complex cascade is the transcriptional regulation of a broad range of physiological activities, including the release of key proinflammatory cytokines such as TNF-α, as well as dictating cellular fate, migration and differentiation.

Initiation of the MAPK pathway is dependent not only on the cell type, but also on the magnitude and duration of stimulation [19]. Importantly, deregulation of MAPK signalling is implicated in a variety of diseases, including cancer. Indeed, one of the master regulators of cellular survival, Ras, is a key MAPKKK involved in ERK activation [16,20]. Ras, a GTPase that has intrinsic kinase activity, is tethered to the plasma membrane by farnesylation and activates the Raf family of protein kinases, which function as MAPKKs to eventually activate ERK [16,20]. Although MAPK activation is generally implicated in cellular survival [17], other studies show that this pathway plays an anti-proliferative role, particularly in regions of hypoxia and in ischemic-reperfusion injuries associated with neuronal and renal damage [19]. The activation of JNK and p38 has also been implicated in mediating the onset of oncosis during hypoxic stimulation of lung epithelial cells [21]. Taken together, these data suggest that the MAPK pathway plays a key role during the early stages of the initiation of oncotic cell death and imply a central role for these pathways during the host cell response to microbial infection and intoxication.

Microbes have evolved ways of subverting normal MAPK activation pathways to perpetuate disease onset and development. Pore-forming toxins (PFT), including α-hemolysin from *Staphylococcus aureus*, and anthrolysin O from *Bacillus anthracis*, are able to activate the p38-MAPK pathway and stimulate the release of IL-8 from intoxicated A549 cells [22]. This activation was shown to be dependent on extracellular divalent cations such as Ca^2+^ [22]. Other studies have shown that *C. perfringens* α-toxin, although not a pore-forming toxin and not related to *C.* *septicum* α-toxin, is capable of inducing the proinflammatory release of cytokines, including TNF-α, IL-6 and IL-1bβ, in an ERK-MAPK dependent manner [23].

The observation that *C*. *septicum* α-toxin is able to induce cellular oncosis in intoxicated nucleated cells, in conjunction with the ability of the MAPK pathway to induce cell death, and the ability of other PFT to activate this pathway, raised the question of whether α-toxin was capable of activating the MAPK pathway*.* Here we have shown that α-toxin is able to activate all three arms of the MAPK pathway, in a dose-dependent manner, and that activation is associated with TNF-α release both *in vitro* and *in vivo.* Activation of ERK was shown to be Ras-c-Raf dependent. By contrast, activation of the JNK and p38 pathways appeared to occur independently of Ras-c-Raf, although the potential for cross-talk between the pathways, especially during an infection, cannot be excluded*.*

## 2. Results

### 2.1. α-Toxin Results in the Activation of the Three Canonical Arms of the MAPK Pathway

**Figure 1:**
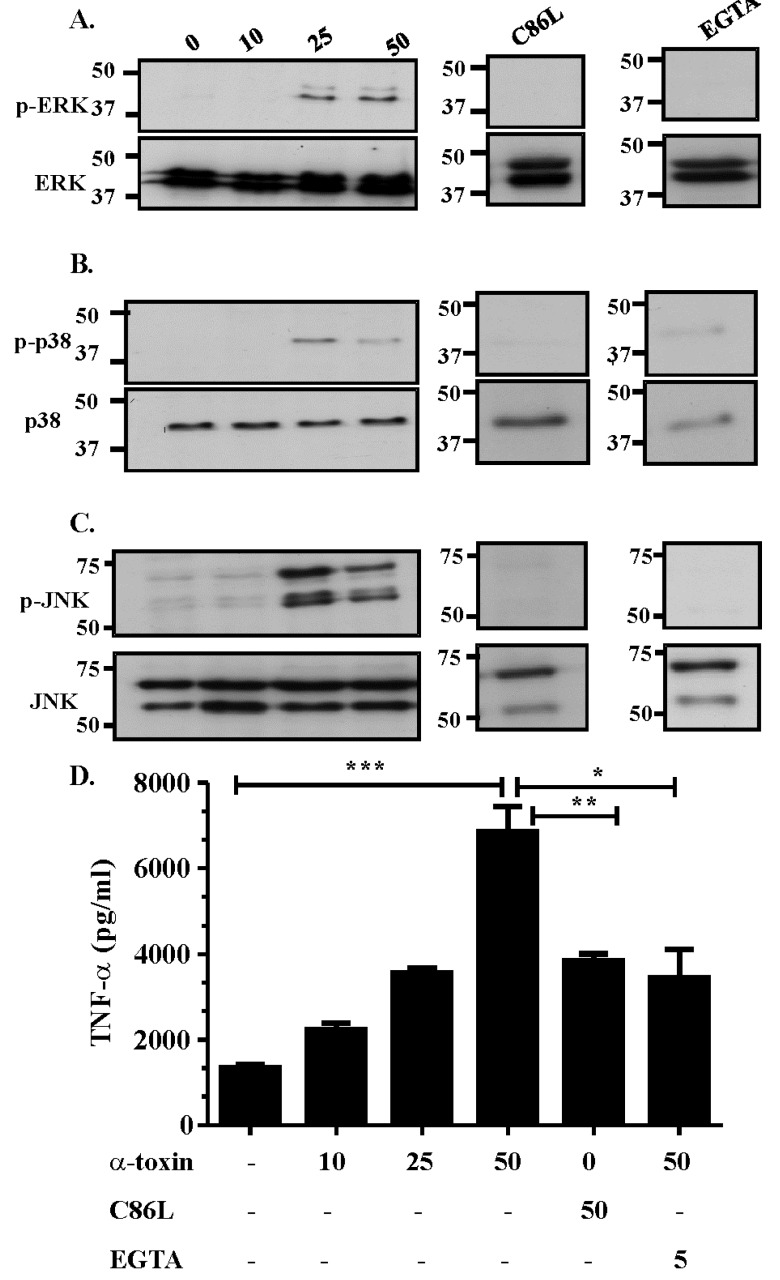
α-toxin intoxication of Vero cells activates all three components of the MAPK pathway. Vero cells were stimulated for 60 min with medium only (0) or with 10, 25 or 50 ng/mL of purified *C. septicum* α-toxin, 50 ng/mL of the purified C86L α-toxin variant or 5 mM EGTA followed by stimulation with 50 ng/mL of purified α-toxin. Cells were then lysed, proteins separated by SDS-PAGE and Western blotted with phospho-ERK or ERK (**A**), phospho-p38 or p38 (**B**), and phospho-JNK or JNK (**C**) specific antibodies; (**D**) Culture supernatants of cells treated with purified α-toxin were collected and levels of TNF-α quantified by ELISA. Values represent the mean ± SEM (*n* = 3, * *p* = 0.0184, ** *p* = 0.0078, *** *p* = 0.0007).

The MAPK pathway is one of the primary pathways activated within cells following contact with bacterial toxins [16]. *C. septicum* α-toxin has previously been shown to be essential for disease [7] and may play an important role in modulating the host’s innate immune response to *C. septicum* infection. To determine if α-toxin modulates the MAPK pathway of intoxicated host cells, Vero cells were treated with various concentrations of purified α-toxin (0–50 ng/mL) for 60 min and Western blots of cell lysates were used to detect phosphorylation of the three subsets of the MAPK pathway (Figure 1). The results showed that treatment of cells with α-toxin resulted in the phosphorylation, and therefore the activation, of ERK, p38 and JNK (Figure 1A–C). This activation was dose-dependent, with 25 to 50 ng/mL of α-toxin capable of inducing activation of all three components. Phosphorylation of p38 appeared to be higher in cells treated with 25 ng/mL of toxin, suggesting that higher concentrations of toxin may reduce α-toxin-dependent, p38-mediated signalling (Figure 1A–C). Culture supernatants isolated from intoxicated cells contained the cytokine TNF-α (Figure 1D), which suggests that nanogram amounts of *C. septicum* α-toxin activates all three components of the MAPK pathway and results in TNF-α release.

### 2.2. Activation of the MAPK Pathway is Dependent on Efficient Receptor Binding, Pore Formation and Extracellular Calcium Influx

The primary means by which PFT damage host cells is by disrupting their membrane stability and osmotic balance [24]. In addition, PFT are capable of modulating the host immune response independently of their pore-forming abilities [22,25]. Previous work has shown that binding of *C. septicum* α-toxin to GPI-anchored proteins, which are its primary receptors, is restricted to loops 1 and 3 and the α-helix in domain 1 [12,26]. A cysteine at position 86 is essential for toxin function and substitution of this cysteine residue with leucine (C86L) resulted in a 7 × 10^6^-fold reduction in receptor binding affinity [12,26]. The resultant inability to form pores abolished both hemolytic activity on blood agar and virulence in a mouse myonecrosis model, providing evidence that efficient receptor binding and pore formation are essential for fulminant disease [14].

To assess the importance of receptor binding and pore formation in α-toxin-mediated MAPK activation, we tested the ability of a purified α-toxin C86L derivative to activate the MAPK pathway in Vero cells. The results showed that cells treated with the C86L-modified protein did not activate the MAPK pathway since no ERK, JNK or p38 phosphorylation was observed in cells treated with 50 ng/mL of this inactive toxin derivative (Figure 1A–C). These data suggest that MAPK activation in α-toxin treated cells *in vitro* is dependent on efficient receptor binding and pore formation. Treatment of cells with the C86L derivative reduced, but did not totally abolish, TNF-α release (Figure 1D), indicating that TNF-α release may be stimulated by an alternative pathway that does not require pore formation or specific receptor binding.

### 2.3. The Role of Extracellular Ca^2+^ in Activation of the MAPK Pathway

The influx of extracellular Ca^2+^ following contact with PFT is often a prerequisite for the efficient activation of signalling pathways [22,27]. Extracellular Ca^2+^ is essential for α-toxin-mediated cell death, with fluctuations in Ca^2+^ levels in intoxicated cells shown to be solely due to the influx of extracellular Ca^2+^ [28]. For this reason, the downstream effects of deregulated Ca^2+^ influx were assessed following α-toxin treatment.

To determine the effect of α-toxin-induced extracellular Ca^2+^ influx on MAPK activation, cells were pre-treated with 10 mM EGTA for 1 h, to chelate any free Ca^2+^, and then treated with 50 ng/mL of purified α-toxin. The results demonstrated that an influx of extracellular Ca^2+^ was critical for α-toxin-mediated MAPK activation since cells pre-treated with EGTA failed to activate the three branches of the MAPK pathway (Figure 1A–C). EGTA treatment also reduced, but did not abolish, TNF-α release (Figure 1D), providing further evidence that MAPK activation is associated with TNF-α release.

### 2.4. Inhibitor Studies Suggest that There Is Cross Talk between the ERK, JNK and p38 Pathways Following α-Toxin Intoxication

**Figure 2:**
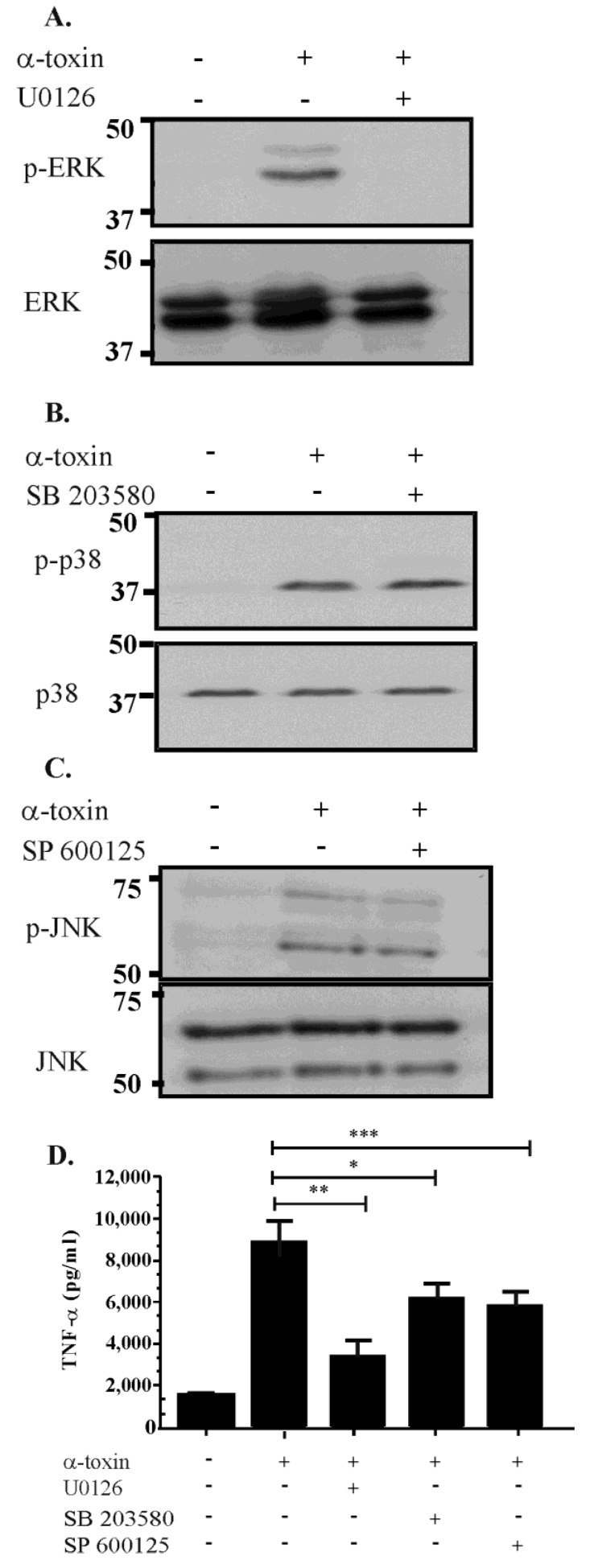
Inhibitor studies suggest that α-toxin-mediated activation of the ERK pathway is MEK1/2 dependent. Cells were pre-treated for 60 min with 10 mM U0126, 50 mM SB203580 or 100 mM SP600125 to inhibit the ERK, p38 and JNK pathways, respectively. Cells were then subsequently stimulated with 50 ng/mL of α-toxin and analysed by Western blotting for activation of all three components of the MAPK pathway (**A**–**C**); Culture supernatants were collected and TNF-α levels quantified by ELISA (**D**). Error bars represent SEM (*n* = 3, * *p* = 0.0219, ** *p* = 0.0019, *** *p* = 0.0139).

Activation of the MAPK pathways can result from different stimuli and by different mechanisms. To determine which pathway is activated by α-toxin, we pre-treated cells with the following inhibitors of each of the three major components of the MAPK pathway: U0126 (a MEK-specific inhibitor), SP600125 (a JNK-specific inhibitor), or SB203580 (a p38-specific inhibitor). Vero cells were incubated with the inhibitors and then treated with 50 ng/mL of purified α-toxin. The results (Figure 2) showed that pre-treatment with U0126 reduced α-toxin-mediated ERK phosphorylation and reduced TNF-α release when compared to cells treated with α-toxin alone (*p* ≤ 0.05) (Figure 2A,D). By contrast, pre-treatment of cells with SP600125 or SB203580 failed to affect the phosphorylation of the p38 or JNK substrates (Figure 2B,C). These inhibitors affect the catalytic activity of JNK and p38, respectively, not the kinase-mediated phosphorylation of these proteins [29]. Although we did not observe a direct effect of these inhibitors on JNK and p38 phosphorylation, cells pre-treated with SP600125 and SB203580 and subsequently treated with α-toxin released lower levels of TNF-α compared to cells stimulated with α-toxin alone (Figure 2D). These data suggest that all three pathways are involved in the host cell’s response to exposure to α-toxin, which is reflected in their capacity to release TNF-α. ERK activation appears to be MEK-dependent, although the kinases responsible for upstream activation of JNK and p38 are not known. The results suggest that no one pathway is responsible for α-toxin-induced TNF-α production.

### 2.5. Activation of the ERK Pathway Is Ras-c-Raf Dependent

The GTPase Ras is one of the major enzymes responsible for ERK activation [16,30]. Ras is a key regulator of cellular survival [30] and is attached to the host cell membrane by farnesylation, as well as by other processes. Ras functions as a super regulator and has the ability to activate other signalling pathways that regulate host cell signal transduction [20]. One of these molecules is c-Raf, a serine/threonine kinase involved in the regulation of numerous physiological activities, including cellular survival [31]. c-Raf kinase activity is tightly regulated at a series of phosphorylation sites, including Ser259 [31]. c-Raf is inactive when phosphorylated at Ser259; when c-Raf is activated, this site is dephosphorylated, thereby blocking its interaction with the regulatory protein 14-3-3 [31].

**Figure 3:**
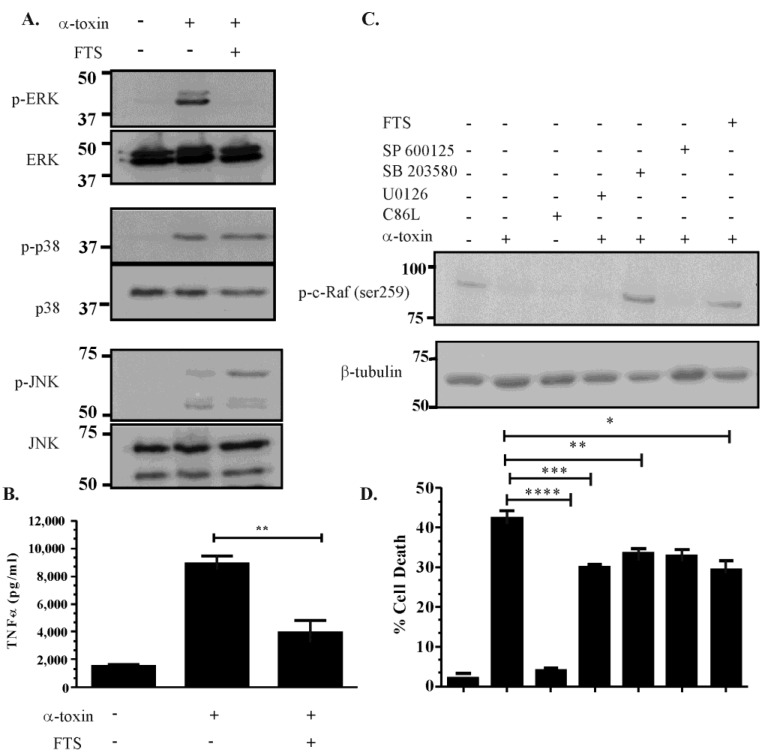
α-toxin mediated MAPK activation occurs in a c-Raf dependent and independent manner**. **Vero cells were pre-treated with 100 mM of FTS for 60 min and subsequently stimulated with 50 ng/mL of purified α-toxin for 60 min. Control cells were either untreated or treated with α-toxin only. Cell lysates then were analysed for their MAPK-activation profile by examining phospho-ERK or ERK**, **phospho-p38 or p38 and phospho-JNK or JNK levels (**A**); Culture supernatant was also isolated from FTS-pre-treated cells and the control cells and analysed for TNF-α release (** *p* = 0.0033) (**B**); Lysates were also isolated from Vero cells stimulated with α-toxin, its C86L derivative, or from cells pre-treated with various inhibitors and subsequently stimulated with α-toxin, and probed for c-Raf activity by detecting c-Raf dephosphorylation at Ser-259, with β-tubulin used as a loading control (**C**); The same treated cells were analysed for cellular viability using an MTT assay (**D**). Error bars represent SEM (*n* = 3, * *p* = 0.016, ** p = 0.0128, *** *p* = 0.0027, **** *p* ≤ 0.0001).

To investigate if α-toxin activates the MEK-ERK pathway in a Ras-c-Raf dependent manner, Vero cells were pre-treated with 100 μM farnesylthiosalicyclic acid (FTS) for 1 h and subsequently stimulated with α-toxin. FTS inhibits the interaction of Ras with cell membranes by disrupting the farnesylation of Ras, thereby blocking the ability of this protein to activate downstream kinases such as c-Raf. The results showed that FTS pre-treatment reduced α-toxin-induced ERK activation, but did not affect p38 or JNK activation (Figure 3A). FTS pre-treatment also reduced α-toxin-induced TNF-α release (Figure 3B), suggesting that α-toxin activation of the MAPK pathway occurs in both a Ras-c-Raf dependent and independent manner. α-toxin intoxication also affected c-Raf phosphorylation, and hence activation, since intoxicated cells had reduced Ser259 phosphorylation when compared to unstimulated cells (Figure 3C). Pre-treatment with U0126 did not affect c-Raf activation (Figure 3C), which suggests that deactivation of MEK does not affect upstream Ras activation, which is consistent with the cascade normally induced during ERK-MAPK activation. By contrast, c-Raf activation was not observed in cells pre-treated with the p38-inhibitor SB203580 (Figure 3C). Cells pre-treated with the JNK inhibitor SP600125 displayed reduced c-Raf activation (Figure 3C), suggesting that α-toxin-mediated activation of JNK may occur via a c-Raf dependent and a c-Raf independent manner. FTS pre-treatment reversed c-Raf phosphorylation (Figure 3C), suggesting that c-Raf is indeed activated primarily in a Ras-dependent manner.

Consistent with the hypothesis that the MAPK pathway contributes to α-toxin-mediated cell death, MTT cytotoxicity assays were performed on α-toxin intoxicated cells and cells pre-treated with the panel of MAPK pathway inhibitors. The results showed that α-toxin was capable of inducing cell death in a process that required efficient receptor binding and pore formation, since stimulation with the α-toxin C86L substitution derivative failed to induce cytotoxicity (Figure 3D). Cells separately pre-treated with inhibitors to the MAPK pathway and subsequently stimulated with α-toxin showed a moderate reduction in cell death (Figure 3D), suggesting that all three components of the MAPK pathway contribute to α-toxin-induced cell death. Taken together, these data suggest that α-toxin-induced MAPK activation occurs in a Ras-c-Raf dependent and independent manner, induces the release of TNF-α, and contributes to cell death. Given these results α-toxin-mediated activation of the MAPK pathway has the potential to play a significant role in *C. septicum*-mediated disease.

### 2.6. The Role of α-Toxin in Vivo Is Multifactorial

**Figure 4:**
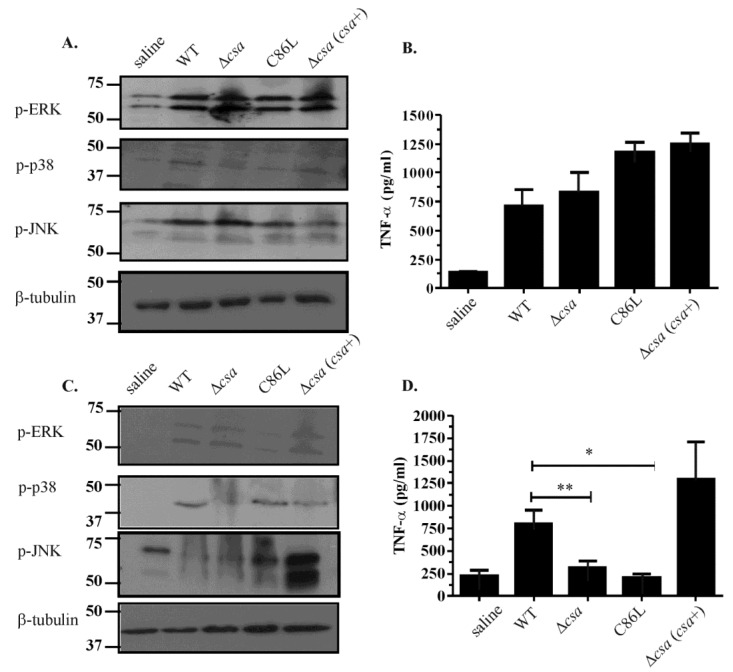
*In vivo* MAPK activation in thigh tissues isolated from mice infected with isogenic *C. septicum* strains. (**A**) Thigh tissues were isolated from mice infected with various strains of *C. septicum* and homogenised in lysis buffer. Lysates were then separated using SDS-PAGE and probed for phospho-ERK, phospho-p38 or phospho-JNK. Samples were also probed using β-tubulin as a loading control; (**B**) Thigh lysates were used to perform ELISA for TNF-α levels in infected tissues; (**C**) Spleens were isolated from mice infected with various strains of *C. septicum* and homogenised in lysis buffer. Lysates were then separated using SDS-PAGE and probed for phospho-ERK, phospho-p38 or phospho-JNK. Samples were also probed using β-tubulin as a loading control; (**D**) Splenic lysates were used to perform ELISA for TNF-α levels in infected tissues. Error bars represent SEM (*n* = 3, * *p* = 0.022, ** *p* = 0.049).

To determine the ability of α-toxin to mediate MAPK activation *in vivo* we analysed tissue lysates obtained from mice infected with an isogenic panel of *C. septicum* strains, including the wild-type *C. septicum* strain JIR6086, an isogenic α-toxin mutant (Δ*csa*), the mutant complemented with the receptor binding mutant (Δ*csa*(*csa*_C86L_) or simply *C86L*) and the mutant complemented with the full length *csa* gene in trans (Δ*csa*(*csa^+^*)). In all strains disease was allowed to progress to the point where euthanasia was required in the wild-type strain, we then examined the infected thigh muscles and the spleens isolated from these mice. Spleen samples were examined to detect systemic dissemination of toxin and distal effects, by looking for MAPK activation and TNF-α release. Analysis of MAPK activation within the thigh tissues revealed pan-activation of all three components, ERK, JNK and p38 (Figure 4A). TNF-α release at the site of infection was not dependent upon the ability of the infecting isolate to produce active α-toxin since the wild-type *C. septicum* strain activated TNF-α release to approximately the same extent as the Δ*csa*, *C86L* and Δ*csa(csa*^+^) strains (Figure 4B).

By contrast, analysis of the splenic lysates isolated from the same mice provided evidence that TNF-α release in the spleen was α-toxin-mediated and that this release was dependent on efficient receptor binding and pore formation (Figure 4D). Mice challenged with the Δ*csa* and *C86L* strains did not elicit the release of TNF-α at a significantly different level when compared to tissues isolated from mice infected with the wild-type and complemented strains (Figure 4D).

Analysis of MAPK activation in these splenic lysates showed pan-activation of the ERK and JNK pathways, irrespective of the infecting strain, suggesting that activation of these pathways at distal sites could be due to other *C. septicum* virulence factors, or bacterial cell fragments that reach and are subsequently cleared by the spleen (Figure 4C). These data showed that infection with *C. septicum* activates and de-regulates host signalling pathways at sites distal to the infection site and does not only affect regions localised to the active infection.

## 3. Discussion

The mechanism by which *C. septicum* interacts with and modulates the host’s innate immune response is not well understood; it is different to the process observed in *C. perfringens* infections, even though both of these necrotic diseases are characterised by a paucity of PMNs at the site of infection [7,32,33]. Vascular leukostasis is not associated with up-regulated cellular adhesion markers such as ICAM, ELAM, PECAM and gpIIbIIIa, as it is in *C. perfringens* infections, suggesting that there is an alternate mechanism by which host cells are modulated to allow bacterial invasion and subsequent intoxication [34,35,36,37,38]. In this report we have shown that *C. septicum* α-toxin is capable of activating the MAPK pathway by the Ras-c-Raf route, providing a mechanism by which this toxin may modulate and regulate cellular fate. Sub-lytic concentrations of α-toxin are sufficient to activate all components of the MAPK pathway, which is important in the broader context of systemic effects of the toxin on the host. Recent studies also have shown that *C. perfringens* α-toxin, which has phospholipase C activity and is not a PFT, activates the MEK/ERK pathway [39,40,41] and it is also clear that sub-lytic levels of PFT from other bacteria activate many cellular pathways, including the MAPK pathway [42]. For example, *C. perfringens* β-toxin induces the phosphorylation of both p38 and JNK [43].

We previously showed that *C. septicum* α-toxin induces programmed cellular oncosis in intoxicated cells, as a consequence of extracellular calcium influx, which leads to activation of the calpain-cathepsin cascade, lysosomal disruption and mitochondrial dysfunction [28]. Unlike many other bacterial toxins, where intoxication results in the induction of either apoptotic or necrotic pathways, cells stimulated with α-toxin appear to display hallmarks of both pathways and undergo cellular oncosis following intoxication. Based on these observations, we postulated that α-toxin-mediated modulation of the host cellular network was a complex process that involved the induction of deregulated cell death by the activation of a network of different, yet interlinked, pathways.

One of the key pathways involved in host cell signalling in response to PFT is the MAPK pathway [17]. Therefore, we decided to determine if treatment of host cells with purified α-toxin activated the MAPK pathway and to determine the potential downstream effects of that activation both *in vitro* and *in vivo.* The results showed that stimulation of Vero cells with purified α-toxin activated all three components of the MAPK pathway (ERK, p38 and JNK) in a dose-dependent manner that was associated with TNF-α release. In addition, analysis of tissues obtained from actual infections highlighted that although α-toxin is the major toxin produced by *C. septicum*, the infectious process is multifactorial and results in effects that are both localised and distal to the initial site of infection.

Activation of the ERK pathway was shown to be MEK-dependent since cells pre-treated with the MEK inhibitor U0126 did not activate ERK. By comparison, cells pre-treated with the p38 inhibitor SB203580 and the JNK inhibitor SP600125 did not affect p38 and JNK phosphorylation, respectively, but did influence TNF-α levels. These inhibitors target the enzymatic activity of p38 and JNK, not their ability to be activated. Furthermore, cells pre-treated with FTS, a Ras-GTPase inhibitor, showed reduced activation of ERK, but unaltered JNK or p38 activation, suggesting that α-toxin activates the ERK-MAPK pathway in a Ras-GTPase dependent manner whereas activation of the p38 and JNK pathways occurs in a Ras-independent manner. α-toxin-treatment also activated c-Raf since Ser259 was dephosphorylated in α-toxin-treated cells. FTS treatment restored c-Raf Ser259 phosphorylation to basal levels, suggesting that c-Raf activation occurs in a Ras-GTPase-dependent manner since Ras is bound to the host cell membrane by farnesylation, and it is this interaction that is affected by FTS treatment. c-Raf activation may either result from cross-talk between p38 and c-Raf by an independent pathway downstream of Ras-mediated c-Raf activation or from cross-inhibition. Cross-interaction and inhibition of c-Raf activation, as determined through the use of SB203580, has previously been described since p38 and c-Raf share a key threonine residue at position 106, which is essential for SB203580-mediated p38 suppression [44]. Therefore, SB203580-mediated inhibition of c-Raf activity may also be due to cross-inhibition between these two pathways.

Finally, *in vivo* studies have shown that mice infected with wild-type *C. septicum*, as well as strains which do not produce α‑toxin (Δ*csa*), or are deficient in pore formation (*C86L*), also activate the MAPK pathways and stimulate the release of TNF-α, but in a manner different to that anticipated. Lysates of thigh muscle tissues extracted from mice infected with the isogenic panel of *C. septicum* strains were able to activate all three MAPK pathways and induce the release of TNF-α at equivalent levels. In a previous study [45] microvascular perfusion deficits caused by *C. septicum* were analysed using intravital microscopy of mouse cremaster muscles stimulated with culture supernatants from the same wild-type and α-toxin mutant strains used here. The results showed that, even in the absence of α-toxin, culture supernatants were capable of inducing vascular deficits [45]. Indeed, only a 60% reduction in vascular permeability was observed in cells treated with the supernatant of the α-toxin mutant in comparison to supernatants from the wild-type, suggesting that at least in localised areas of infection, α‑toxin may work synergistically with other virulence factors to establish and allow the spread of infection and disease [45]. Other studies have shown that A549 cells stimulated with a derivative of pneumolysin that has altered receptor binding exhibits decreased levels of IL-8 production [22], while cholesterol dependent cytolysins, such as *C. perfringens* perfringolysin O and *B.* *anthracis* anthrolysin O, are able to activate TLR-4 independently of their pore forming abilities [25]. Furthermore, the presence of various bacterial by-products, such as peptidoglycan and lipotechoic acid, are also likely to act as inducers of the MAPK pathway [46,47], by alternative signalling pathways perhaps involving Nod-like or Toll-like receptors, which are known to feed into the MAPK signalling cascades [48,49]. In the current study splenic lysates from mice infected with the wild-type *C*. *septicum* strain displayed higher levels of TNF-α release than the equivalent extracts from mice infected with the α-toxin mutant. This difference was reversed upon complementation with the wild-type *csa* gene. No bacteria were recovered from the spleens of any of the infected mice, suggesting that TNF-α release most likely occurred as a result of systemic toxin release. These data supported our *in vitro* data, which showed that TNF-α release occurred in an α-toxin dependent manner. Despite *C. septicum* disease being thought of as a localised infection, α-toxin clearly is able to be disseminated within a short time period (3–4 h post-infection) and thereby interact with and modulate the host’s innate immune response. These data further highlight the importance of the early stages of host-pathogen interactions and suggest that during a *C. septicum* infection, systemic dispersal of toxin has the potential to prime and condition the immune response to induce a septic shock-like phenomenon whereby infection- and toxin- induced pro-inflammatory cytokine release exacerbates the developing infection.

TNF-α has been shown to be one of the primary proinflammatory cytokines released by leucocytes following systemic trauma [50]. The release of TNF-α varies, depending on the nature of the stimulation and the balance of the inflammatory/anti-inflammatory cytokines that are present. That study also showed that LPS-induced TNF-α production was not directly associated with p38 activation [50]. Furthermore, these data highlight that activation of the MAPK pathway during systemic infection does not necessarily correlate with cytokine release since the MAPK pathway interacts with a multitude of other signalling pathways *in vivo*. Therefore, cytokine release may be the result of MAPK-activation of these alternative pathways [50]. These data mirror our findings since analysis of thigh tissues isolated from *C. septicum-*infected mice revealed MAPK activation, irrespective of the strain used. No direct effect of α-toxin on TNF-α release was observed in thigh tissues, which represented the localised site of infection. However, analysis of the distal site of infection, which was representative of systemic dispersal, suggested that TNF-α may potentially play an important role in α-toxin-induced pathogenesis and cell death *in vivo.* These data provide evidence for *C. septicum* α-toxin-mediated activation of the MAPK pathway and TNF-α release, and suggest an important link between host cell signalling pathways and cell death. We propose a model for disease in which *C. septicum* α-toxin activates all three arms of the canonical MAPK pathway in a calcium dependent and Ras-c-Raf dependent and independent manner. The kinase upstream of JNK and p38 activation was not identified and all three pathways are most likely to interact and modulate one another. The release of the pleiotropic cytokine TNF-α is also induced by α-toxin and in association with the MAPK pathway may contribute to the oncotic phenotype seen during *C. septicum* intoxication *in vitro* as well as the paucity of leucocytes seen *in vivo.*

In summary, *C. septicum* strains that did not encode a functional α-toxin did not induce high levels of TNF-α release at distal sites of infection, whereas strains that produced functional toxin were able to induce TNF-α release at comparable levels to that seen in a localised area of infection. Furthermore, the concentration of α-toxin at distal sites of infection is likely to be at sub-lytic levels, at least initially, while the localised area of infection will have larger amounts of toxin. Variations in toxin levels may directly impact disease progression *in vivo* as the infected region will have signs of cell and tissue destruction, while sub-lytic levels of toxin may induce both cell death and contribute to the characteristic paucity of leucocytes seen during *C. septicum* infection *in vivo.* Sub-lytic toxin levels may also induce the release of various pro-inflammatory cytokines, which may exacerbate the host inflammatory response following infection.

## 4. Materials and Methods

### 4.1. Bacterial Strains and Growth Conditions

*C. septicum* was grown on heart infusion (HI) media (Oxoid, Hampshire, UK) supplemented with 0.005% (*w*/*v*) L-cysteine (Sigma Aldrich, St. Louis, MO, USA) and 0.38% (*w*/*v*) glucose (HIS). Prior to inoculation broth media were boiled for 10 min to render them anaerobic. Agar cultures were grown at 37 °C under anaerobic conditions (10% (*v*/*v*) H_2_, 10% (*v*/*v*) CO_2_ in N_2_). The *C. septicum* strains used for virulence studies included the wild-type *C. septicum* strain JIR6086, its isogenic *csa::erm* (B) mutant JIR6111, JIR6146 (JIR6111 complemented with a shuttle plasmid carrying the wild-type *csa* gene), and JIR6256 (JIR6111 complemented with a shuttle plasmid carrying the *csa_C86L_* gene) [14]. Strains were grown on HIS agar supplemented with the relevant antibiotics, as previously described [14] and were assessed for cytolytic activity by plating onto horse blood agar [32].

### 4.2. Protein Purification

Expression vectors encoding histidine-tagged α-toxin or a C86L substitution derivative of α-toxin were a gift from R.K Tweten (University of Oklahoma, Norman, OK, USA). These proteins were purified by the Monash University Protein Production Unit using Ni^2+^ affinity and cation exchange columns, as previously described [51].

### 4.3. Toxin Stimulation of Vero Cells

Vero cells were maintained in MEM media (Invitrogen, Carlsbad, CA, USA) supplemented with 10% (*v*/*v*) heat inactivated fetal calf serum (MultiSer, Cytosystems, Castle Hill, Australia). The cells (1 × 10^6^) were dispensed into 24-well tissue culture plates (Sarsdsedt, Numbrecht, Germany), grown to 80%–90% confluence and serum-starved for 24 h in MEM medium alone prior to treatment. These monolayers then were treated with various concentrations of purified α-toxin for 1 h before being analysed by Western blotting. When required for inhibitor studies, cells were pre-treated for 1 h with either 10 μM U0126 (Cell Signalling Technology, Danvers, MA, USA), 100 μM SB203580 (Merck, Whitehouse Station, NJ, USA), 50 μM SP600125 (Merck, Whitehouse Station, NJ, USA), 100 μM farnesylthiosalicyclic acid (FTS) (Santa Cruz Biotechnology, Dallas, TX, USA) or 5 mM EGTA (Sigma Aldrich, St. Louis, MO, USA) before stimulation with purified toxin (50 ng/mL) for 1 h. TNF-α release was measured using a TNF-α ELISA Kit (Invitrogen, Carlsbad, CA, USA).

### 4.4. Cell Cytotoxicity Assays

Vero cells were grown to confluence and seeded into 96-well plates (1 × 10^5^ cells/mL). Monolayers were treated with medium only (control), 50 ng/mL of purified α-toxin, or its C86L derivative, for 1 h at 37 °C under 5% (*v*/*v*) CO_2_. When inhibitors were used, cells were pre-treated for 1 h prior to stimulation with 50 ng/mL of purified α-toxin. Cellular viability was determined using an MTT assay as previously described [52]. Briefly, cells were incubated with 20 μL of 0.5% (*w*/*v*) 3-(4, 5-dimethylthiazol-2-yl)-2, 5-diphenyltetrazolium bromide (MTT; Sigma Aldrich, St. Louis, MO, USA) for 4 h at 37 °C under 5% CO_2_ with gentle shaking. Culture medium then was removed and the insoluble formazan dye was extracted using 200 μL of dimethyl sulfoxide (DMSO) (Sigma Aldrich, St. Louis, MO, USA). The absorbance was then measured using a Tecan (Maennedorf, Switzerland) Infinite M200 plate reader at 570 nm. Percentage cell death was calculated after subtracting the absorbance from cells stimulated with medium only from the absorbance of treated cells.

### 4.5. Western Blot Analysis

Stimulated cells were lysed by boiling in 2× SDS-PAGE sample buffer [28]. To analyse mice infected with *C. septicum*, muscle and splenic tissues were homogenised in lysis buffer (PBS containing 10% (*v*/*v*) Triton-X100) with added protease inhibitor tablets (leupeptin, aprotinin, pepstatin) (Roche, Basel, Switzerland) for 30 min on ice (one tablet in 50 mL of lysis buffer). Lysates were clarified by centrifugation at 12,000× *g* for 10 min at 4 °C, and supernatants were collected for subsequent Western blotting. Protein concentration was determined using a BCA assay (Pierce, Rockford, IL, USA) and 150 μg of protein was loaded per well. Samples were separated on 10% SDS-PAGE gels and transferred onto nitrocellulose membranes (Whatman, Kent, UK). Membranes then were probed using antibodies for phospho-ERK, phospho-p38, phospho-JNK, ERK, JNK, p38, phospho c-Raf (ser259) or β-tubulin (Cell Signalling Technology, Danvers, MA, USA). Membranes were stripped in 25 mM Tris-HCl buffer pH 6.5, containing 10% (*w*/*v*) SDS and 50 mM β-mercaptoethanol and re-probed or duplicate samples were separated by electrophoresis to assay for protein loading.

### 4.6. Virulence Trials

The wild-type *C. septicum* strain and its various mutant derivatives were virulence tested in a mouse myonecrosis model as previously described [7,14]. Briefly, *C. septicum* strains were grown under appropriate antibiotic selection to obtain pure cultures, used to inoculate 20 mL of HI broth supplemented with 0.005% L-cysteine and 0.02% glucose, and then incubated for 6 h at 37 °C under anaerobic conditions or until an OD_600_ of 0.9–1.2 was obtained. These cultures were used to inoculate 90 mL of the same medium and grown until an OD_600_ of 2.0–2.2 was obtained. The cells were then washed once in Dulbecco’s modified phosphate buffered saline (DPBS) and resuspended in three times the cell pellet volume, which was equivalent to ca. 10^9^ colony forming units. Female BALB/c mice were injected into the right hind thigh with 50 μL of washed cells and monitored for 12 h for the characteristic signs of disease, including limping, swelling of the footpad and thigh, blackening of the footpad and thigh [7,14]. Thigh and spleens were collected immediately following sacrifice and placed onto ice. Mouse virulence trials were conducted under a protocol that was approved by the Monash University MARP 2 Animal Ethics Committee (Project Number E/0614/2007/M), in strict accordance with legally binding Victorian State Government regulations. All efforts were made to minimize suffering.

### 4.7. Statistical Analysis

Statistical analysis was carried out using an unpaired Student’s t test with GraphPad Prism software. All error bars are shown as mean ± SEM, unless otherwise stated *n* = 3.
